# Genetic variant in visfatin gene promoter contributes to reduced risk of hepatocellular carcinoma in a Chinese population

**DOI:** 10.18632/oncotarget.12864

**Published:** 2016-10-25

**Authors:** Zhitong Wu, Yifan Sun, Yiyong Huang, Shengbo Zhu, Yi Feng, Huifen Ye, Chunming Liu, Shifu Tang

**Affiliations:** ^1^ Department of Clinical Laboratory, Eighth Affiliated Hospital of Guangxi Medical University, Guigang City People's Hospital, Guigang, Guangxi, China; ^2^ Department of Clinical Laboratory, Third Affiliated Hospital of Guangxi University of Chinese Medicine, Liuzhou, Guangxi, China

**Keywords:** visfatin, polymorphism, hepatocellular carcinoma

## Abstract

Knowledge on the role of gene variants in the visfatin promoter region in the hepatitis B virus (HBV)-related liver diseases is limited. In this study, we genotyped two potentially functional single nucleotide polymorphisms (SNPs) in the visfatin promoter region, -1535C>T (rs61330082) and -3187G>A (rs11977021), in 120 HBV-related chronic hepatitis B (CHB) patients, 140 HBV-related liver cirrhosis (HBV-LC) patients, 243 HBV-related hepatocellular carcinoma (HBV-HCC) patients, and 224 asymptomatic HBV carriers. Odds ratios (ORs) with 95% confidence intervals (CIs) were calculated by logistic regression. The results showed subjects with a TT genotype of -1535C>T had a significantly decreased risk of HBV-HCC related to the CC and CC + CT genotypes (adjusted OR = 0.493, 95% CI = 0.313-0.778; OR = 0.535, 95% CI = 0.362-0.791, respectively). A lowered risk also appeared in the comparison between allele T and allele C (OR = 0.734, 95%, CI = 0.581-0.950). However, these associations existed only in people with Zhuang ethnicity, but not in people with Han ethnicity. There were no significant associations between -3187G>A polymorphisms and the risk of HBV-related liver diseases. Our results suggested that visfatin -1535C>T polymorphisms might be associated with decreased risk of HBV-HCC among the ethnic Zhuang population in Guangxi, China.

## INTRODUCTION

Hepatocellular carcinoma (HCC) is a common type of liver cancer with high morbidity and mortality, and it is estimated that about 466,100 new cases happened in China, 2015, and about 422,100 Chinese died from HCC [1]. HCC is a multifactorial disease, with hepatitis B virus (HBV) and C (HCV) infections, aflatoxin-B1 (AFB1) exposure, and excessive alcohol intake considered to be the most prominent etiologies linked to the development of HCC [2, 3]. Over the last two decades, the rising incidence of HCC has been related to the burgeoning incidence of obesity, nonalcoholic fatty liver disease (NAFLD), and metabolic syndrome [4]. However, the accurate etiology of HCC remains elusive; for example, the mechanisms of genomic alterations in HCC require examination [5].

Frequently, HCC development is associated with the induction of inflammation [6, 7]. Genetic mutations in inflammation-related genes, especially in cytokines, may be involved in the progression of HCC. Recent studies have suggested that mutations in cytokines, such as tumor necrosis factor-α (TNF-α), interferon gamma (IFN-γ), interleukin-18 (IL-18) [8], IL-6 [9], IL-4 [10], and IL-17 [11], were associated with the risk of HCC. Visfatin—also called nicotinamide phosphoribosyltransferase (NAMPT), pre-B-cell colony-enhancing factor (PBEF)—is a common adipocytokine released by the adipose tissue. Visfatin is upregulated during adipocyte differentiation and involved in the pathogenesis of NAFLD [12], regulates cell energy balance in chronic hepatitis [13]. Elevated visfatin expression has been reported in various digestive system cancers [14–16].

The visfatin gene, located on chromosome 7q22, spans an approximate length of 34.7 kbp and exhibits 11 exons and 10 introns [17]. Several frequent polymorphisms of the visfatin gene were reported to be associated with low-grade inflammation, type 2 diabetes, obesity, and coronary artery disease [18–20]. Of the single nucleotide polymorphisms (SNPs) in the visfatin promoter region, the -1535C>T (rs61330082) and -3187G>A (rs11977021) loci have been widely studied as disease contributors, because they regulate the expression of the visfatin gene [21, 22]. In fact, recent genetic studies have revealed that polymorphisms of -1535C>T were associated with both increased bladder cancer risk [23] and esophageal squamous cell carcinoma risk [24]. Nevertheless, the potential role of visfatin gene polymorphisms in HCC development remains unknown, and no genome-wide association studies (GWASs) have addressed these SNPs in HCC patients. We hypothesized that the visfatin gene polymorphisms may be associated with risk of HCC, and carried out a case-control study to investigate the association between the -1535C>T and -3187G>A polymorphisms and HCC risk.

## RESULTS

### Demographic characteristics

The basic characteristics of the study population are shown in Table 1. Significant differences were found between the patients and controls in terms of gender, age, ethnicity, and drinking status (*P*-value < 0.05). The number of females was significantly lower among the HBV-HCC patients compared with the control group (8.2% versus 44.2%, *P* < 0.001). The CHB patients were, on average, 10 years younger than the HBV-LC, HBV-HCC patients, and the control group. No significant differences in BMI or smoking were identified across the study groups.

**Table 1:** Characteristics of the study populations

	Control (N=224)	CHB (N=120)	LC (N=140)	HCC (N=243)	*P*
Gender, %(N)					
Male	55.8 (125)	76.7(92)	76.4(107)	91.8(223)	<0.001
Female	44.2(99)	23.3(28)	23.6(33)	8.2(20)	
Age (years), Mean±SD	46.7±7.04	37.0±12.0	48.4±11.94	49.4±11.2	<0.001
<50	60.7(136)	84.1(101)	55.0(77)	55.1(134)	<0.001
≥50	39.3(88)	15.8(19)	45.0(63)	44.9(109)	
Ethnicity, %(N)					
Han	42.4(95)	61.7(74)	61.4(86)	56.8(138)	0.002
Zhuang	51.8(116)	33.3(40)	36.4(51)	40.3(98)	
Other	6.0(13)	5.0(6)	2.1(3)	2.9(7)	
BMI(kg/m^2^), Mean±SD	22.4±3.5	22.1±3.40	22.9±3.6	22.1±3.4	0.058
<25 kg/m^2^,%(N)	82.6(185)	84.1(101)	74.3(104)	84.8(206)	0.062
≥25 kg/m^2^,%(N)	17.4(39)	15.8(19)	25.7(36)	15.2(37)	
Smoking, %(N)					
Yes	32.1(72)	40.8(49)	42.1(59)	35.0(85)	0.143
No	67.9(152)	59.2(71)	57.9(81)	65.0(158)	
Dinking, %(N)					
Yes	28.1(63)	45.0(54)	27.9(39)	34.6(84)	0.007
No	72.9(161)	55.0(66)	72.1(101)	65.4(159)	

CHB, chronic hepatitis B; LC, liver cirrhosis; HCC, hepatocellular carcinoma; SD, standard deviation; Qualitative characteristics: Chi-squared test; Continuous variables: one-way ANOVA test.

### Association of visfatin polymorphisms with the risk of HBV-related liver diseases

The genotype and allele frequencies of visfatin -1535C>T and -3187G>A polymorphisms between the patients and the control subjects are shown in Table 2. All loci did not display deviation from HWE in either the patient or the control groups (*χ^2^* = 0.365, *P* = 0.546 for -1535C>T; *χ^2^* = 1.789, *P* = 0.181 for -3187G>A), suggesting that the subjects used in our study were chosen randomly from the population.

**Table 2:** Association between Visfatin polymorphisms and risk of CHB, LC, HCC

Model	Control N%	CHB vs. Control			LC vs. Control			HCC vs. Control		
		N%	OR(95%CI)*	P*	N%	OR(95%CI)*	*P*	N%	OR(95%CI)*	*P*
−1535C>T										
CC	89(39.7)	52(43.3)	1^ref^		63(45.0)	1^ref^		102(42.0)	1^ref^	
CT	101(45.1)	46(38.3)	0.847(0.509-1.409)	0.522	61(43.6)	0.771(0.511-1.162)	0.214	115(47.3)	0.864(0.587-1.272)	0.458
TT	34(15.2)	22(18.3)	0.956(0.540-1.693)	0.878	16(11.4)	0.609(0.373-0.994)	0.047	26(10.7)	0.493(0.313-0.778)	0.002
Dominant model	135(60.3)	68(56.6)	0.872(0.534-1.422)	0.582	77(55.0)	0.729(0.491-1.081)	0.116	141(58.0)	0.765(0.527-1.109)	0.157
Recessive model			1.040(0.642-1.686)	0.872		0.665(0.430-1.028)	0.066		0.535(0.362-0.791)	0.002
C	279(62.3)	150(62.0)	1^ref^		187(66.8)	1^ref^		319(65.6)	1^ref^	
T	169(37.7)	90(38.0)	0.942(0.682-1.303	0.720	93(33.2)	0.772(0.590-1.009)	0.058	167(34.4)	0.734(0.581-0.950)	0.018
−3187G>A										
CC	52(23.2)	23(19.2)	1^ref^		32(22.9)	1^ref^		50(20.6)	1^ref^	
CT	122(54.5)	72(60.0)	1.685(0.900-3.152)	0.103	84(60.0)	1.150(0.698-1.895)	0.583	144(59.2)	1.404(0.880-2.240)	0.154
TT	50(22.3)	25(20.8)	1.318(0.676-2.571)	0.417	24(17.1)	0.820(0.478-1.410)	0.474	49(20.2)	1.116(0.678-1.835)	0.665
Dominant model	172(76.8)	103(85.8)	1.549(0.843-2.845)	0.159	108(77.1)	1.062(0.650-1.734)	0.810	193(79.4)	1.330(0.842-2.099)	0.221
Recessive model	222(49.6)	134(55.8)	0.916(0.572-1.467)	0.715	132(46.8)	0.737(0.505-1.075)	0.113		0.861(0.611-1.213)	0.392
C	226(50.4)	118(49.2)	1^ref^		148(52.8)	1^ref^		244(50.2)	1^ref^	
T	222(49.6)	122(50.8)	1.137(0.820-1.575)	0.441	132(47.2)	0.931(0.714-1.214)	0.598	242(49.8)	1.053(0.823-1.346)	0.682

* Adjusted for age, gender, BMI, ethnicity, smoking and drinking; ref, reference

**Figure 1:**
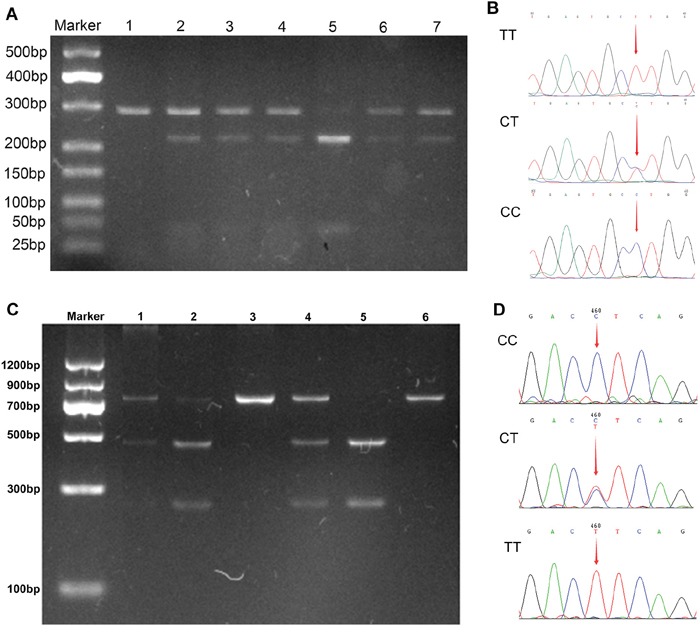
PCR-RFLP and sequencing assay for analyzing the -1535C>T and -3187G>A polymorphisms **A.** PCR-RFLP assay for -1535C>T, lane 1: TT(283bp); lane 2, 3, 4, 6, 7: CT (283bp, 218 bp, 65 bp); lane 5: CC (218bp, 65 bp). **B.** Sequencing assay for -1535C>T, the arrays in panels shown TT, CT, and CC, respectively. **C.** PCR-RFLP assay for -3187G>A, lane 1, 2, 4: CT (731bp, 468bp, 263bp); lane 3, 6: CC(731bp); lane 5: TT (468bp, 263bp). **D.** Sequencing assay for -1535C>T, the arrays in panels shown CC, CT, and TT, respectively.

Regarding visfatin -1535C>T polymorphism, TT, CT, and CC genotypes were found in both the patients and control subjects (Figure 1A, 1B). Although the prevalence of the TT genotype was lower in the HBV-LC (11.4%) and HBV-HCC (10.7%) patients when compared with the control group (15.2%), the distribution of the visfatin genotype and allele frequencies did not differ significantly between the patient and the control groups (*P* > 0.05). Results from logistic regressive analyses, adjusted for confounding factors, are shown in Table 2. Significant associations were observed between the visfatin -1535C>T polymorphism and HBV-HCC risk in the co-dominant model (TT versus CC: OR = 0.493, 95% CI = 0.313-0.778, *P* = 0.002), the recessive model (TT versus CT + CC: OR = 0.535, 95% CI = 0.362-0.791, *P* = 0.002), and the allelic model (allele T versus allele C: OR = 0.734, 95% CI = 0.58-0.950, *P* = 0.018).

In addition, significant associations between visfatin -1535C>T polymorphism and HBV-LC risk were presented in a co-dominant model (TT versus CC: OR = 0.609, 95% CI = 0.373-0.994, *P* = 0.047). However, we did not observe significant associations between the visfatin-1535C>T polymorphisms and CHB risk under any comparison models (all *P* > 0.05, Table 2). The analysis of intermediate traits was further performed using CHB and LC samples combined, and no significant associations were observed (all *P* > 0.05, data not shown).

With visfatin -3187G>A polymorphism, three genotypes (TT, CT, and CC, as shown in Figure 1C, 1D) were successfully identified in both the patient and control groups. Genotype and allele analysis found no significant differences within the genotype distribution across the study groups (*P* = 0.345), or in the allele distribution (*P* = 0.217). Logistic regression analysis, adjusted for gender, age, ethnicity, and drinking status, showed that no single genotype or allele of visfatin -3187G>A loci was associated with an altered risk for CHB, HBV-LC, or HBV-HCC (all *P* > 0.05; see Table 2).

Considering the significant differences of distribution for gender, age, ethnicity, and drinking status, genotype distributions of the two SNPs in different groups stratified by those patient characteristics were also analyzed. According to Table 1, gender was stratified between male and female groups; age was stratified by < 50 and ≥ 50 groups; ethnicity was stratified between Zhuang and Han groups; BMI was stratified as < 25 kg/m^2^ and ≥ 25 kg/m^2^ groups; and the smoking and drinking statuses were stratified between the “Yes” and “No” groups. Interestingly, the association between visfatin -1535C>T polymorphisms and HBV-HCC risk was found in the Zhuang ethnicity population, but not in the Han ethnicity population (Table 3). Statistically, the visfatin -1535C>T TT genotype was associated with a decreased risk of HCC compared with the CC/CT genotypes among the Zhuang ethnicity population (TT versus CC: OR = 0.400, 95% CI= 0.208-0.770, *P* = 0.006; TT versus CT + CC: OR = 0.440, 95% CI = 0.249-0.775, *P* = 0.004). In addition, allele T revealed a decreased HBV-HCC risk compared with allele C (OR = 0.675, 95% CI = 0.47-0.968, *P* = 0.033). However, no significant associations were observed within the subgroups analyzed by age, BMI, smoking, or drinking statuses for the two SNPs (data not shown).

**Table 3:** Stratified effects of Visfatin -1535C>T Polymorphisms on HCC risk estimated by ethnicity

Ethnicity	Control, N(%)	HCC		
		N(%)	OR(95%CI)*	*P*
*Han*	HWE=0.730			
CC	34(35.8)	57(41.3)	1^ref^	
CT	47(49.5)	66(47.8)	0.852(0.480-1.511)	0.583
TT	14(14.7)	15(10.9)	0.651(0.328-1.289)	0.218
Dominant model			0.804(0.463-1.395)	0.437
Recessive model			0.713(0.397-1.281)	0.257
C	115(60.5)	180(65.2)	1^ref^	
T	75(39.5)	96(34.8)	0.827(0.577-1.183)	0.298
*Zhuang*	HWE=0.223			
CC	49(42.2)	43(43.9)	1^ref^	
CT	48(41.4)	44(44.9)	0.893(0.513-1.553)	0.688
TT	19(16.4)	11(11.2)	0.400(0.208-0.770)	0.006
Dominant model			0.729(0.428-1.243)	0.246
Recessive model			0.440(0.249-0.775)	0.004
C	146(62.9)	130(66.3)	1^ref^	
T	86(37.1)	66(33.7)	0.675(0.471-0.968)	0.033

### Haplotype analysis

The LD between the two SNPs within the three comparisons was very weak in both patient and control groups (all *D*^’^ < 0.2 and *r^2^* < 0.01). The value of the lowest frequency threshold (LFT) for haplotype analysis is 0.03, and haplotypes with a frequency less than 0.03 were not considered in the analysis. Finally, four haplotypes (CC, CT, TC, and TT) were constructed according to the observed genotypes of the two SNPs. The frequency of the four haplotypes of the two SNPs are shown in Table 4. The frequency of TC and TT haplotypes was lower than that of CC and CT both in patients and controls; however, the frequency of CC, CT, TC and TT did not differ significantly between patients and controls (*P* > 0.05). Based on the statistical results, we did not observe significant associations between the four haplotypes and risk of HBV-related liver diseases (all *P* > 0.05, Table 4).

**Table 4:** Haplotype analysis between -1535C>T and -3187G>A

Haplotype	N(frequency)				CHB vs Control		LC vs Control		HCC vs Control	
	Control(%)	CHB(%)	LC(%)	HCC(%)	*P*	OR(95%CI)	*P*	OR(95%CI)	*P*	OR(95%CI)
C C	144(32.2)	63(26.4)	102(36.4)	161(33.1)	0.113	0.76(0.53-1.07)	0.243	1.21(0.88-1.65)	0.779	1.04(0.79-1.37)
C T	135(30.1)	87(36.1)	85(30.4)	158(32.6)	0.106	1.31(0.94-1.83)	0.931	1.01(0.73-1.40)	0.411	1.12(0.85-1.48)
T C	82(18.2)	43(17.8)	46(16.4)	83(17.1)	0.880	0.97(0.64-1.46)	0.535	0.88(0.59-1.31)	0.659	0.93(0.66-1.30)
T T	87(19.5)	47(19.7)	47(16.8)	84(17.2)	0.939	10.2(0.68-1.51)	0.358	0.83(0.56-1.23)	0.372	0.86(0.62-1.20)

### Genotype and allele frequencies among different population

To observe the differences among genotype and allele frequencies of visfatin -1535C>T and -3187G>A for different populations, we compared our results with those in different races according to the Haplotype Map (HapMap) project (http://www.ncbi.nlm.nih.gov/snp/). Because no data from different races was found in HapMap for -1535C>T, we compared the genotype and allele frequencies of this SNP with those of previous studies. As shown in Table 5, the genotype and allele frequencies of -1535C>T in this study were significantly different than that of previous studies from China (Jinan province) [24] (*P* < 0.001) and America [25] (*P* < 0.001). There were significant differences in the allele frequencies of -1535C>T in this study compared to those of Singapore's population (*P* = 0.027) [26], but not in genotype frequencies (*P* = 0.065). Statistically, when compared with this study, the genotype and allele frequencies of -3187G>A were found to be similar with the HCB (Han Chinese in Beijing, Asia) ethnicity and the JPT ethnicity (Japanese in Tokyo, Asia), but significantly different from the CEU ethnicity (Utah residents with Northern and Western European ancestry, Europe) and the YRI ethnicity (Yoruba in Ibadan, Africa). The frequency of TT found in our study (22.3%) was significantly higher than those found in the CEU (8.8%) and YRI (0%) populations, but similar to the HCB (20.9%) and the JPT (24.4%) populations. On the other hand, the occurrence of the -3187G>A CC genotype in this study was 23.2%, which was significantly lower than the levels found in the CEU and YRI populations (55.8% and 91.2%, respectively).

**Table 5:** Genotype and allele frequencies among different population

SNPs	Number	Genotype Frequency, N(%)			*P*	Allele frequency, N(%)		*P*
		TT	CT	CC		T	C	
**-1535C>T**								
This study	224	34(15.2)	101(45.1)	89(39.7)		169(37.7)	279(62.3)	
China(Jinan) [31]	405	123(30.4)	216(53.3)	66(16.3)	<0.001	462(57.0)	348(43.0)	<0.001
American [34]	530	45(8)	185(35)	300(57)	<0.001	275(26.0)	785(74.0)	<0.001
Singapore [35]	243	47(19.5)	124(51.0)	72(29.5)	0.065	218(44.9)	268(55.1)	0.027
**-3187G>A**								
This study	224	50(22.3)	122(54.5)	52(23.2)		222(49.6)	226(50.4)	
HapMap-CEU	226	20(8.8)	80(35.4)	126(55.8)	<0.001	120(26.5)	332(73.5)	<0.001
HapMap-HCB	86	18(20.9)	40(46.5)	28(32.6)	0.233	76(44.2)	96(55.9)	0.252
HapMap-JPT	172	42(24.4)	90(52.3)	40(23.2)	0.875	174(50.6)	170(49.4)	0.727
HapMap-YRI	226	0(0)	20(8.8)	206(91.2)	<0.001	20(4.4)	432(95.6)	<0.001

## DISCUSSION

Guangxi, a multiracial province in southern China, possesses one of the highest rates of HCC in the world, and the leading cause of HCC is the high rates of HBV infection in this region [27]. Over the past decade, studies have demonstrated that genetic variation were related to the risk of HBV-HCC for the Guangxi population [9-11, 28, 29]. In this study, we identified that the visfatin -1535C>T polymorphism was associated with HBV-HCC risk. Our data suggested that HBV-infected individuals who carry the visfatin-1535C>T TT genotype may have a lower HCC risk compared with the C-carriers (CC or CT genotypes), especially among those with Zhuang ethnicity.

Our results were consistent with a previous study that examined the association between -1535C>T polymorphisms and esophageal squamous cell carcinoma (ESCC) risk within a Chinese population [24]. In that study, Zhang *et al.* found that the TT and CT genotypes of -1535C>T demonstrated significantly decreased ESCC risk when compared with the CC genotype (OR = 0.33, *P* < 0.01; OR = 0.47, *P* < 0.01, respectively), and also showed a decrease when comparing the T and C alleles (OR = 0.48, *P* < 0.01) [24]. In another study of 407 bladder cancer patients in southwest China, results indicated that the -1535C>T C allele significantly increased bladder cancer risk when compared with the T allele (OR = 1.24, *P* = 0.047). Furthermore, in their study, trends of association within a co-dominant model, dominant model, and recessive model were evident but did not achieve significance; however, there seemed to be a trend toward the incidence of the CC genotype among patients when compared to controls (25.8% in patients and 19.9% in controls) [23]. In addition, subjects with the variant genotypes, CT or TT, also reported reduced risk of CAD, acute respiratory distress syndrome, sepsis, and pneumonia [20, 25, 30].

The -1535C>T loci is located in the promoter region of the visfatin gene, and the polymorphisms of this SNP, which transition C to T, might influence the structure or function of the visfatin protein and the visfatin expression in humans. Ye *et al.* [22] performed a transient luciferase reporter gene assay by transfecting a C variant or a T variant pGL3 basic vector into human lung microvascular endothelial cells (HMVECs), and found the T variant from this SNP resulted in a significant decrease in the transcription rate (1.8-fold; *P* < 0.01). Using the same method, Wang *et al*. also observed the T variant induced nearly a 1.5 fold decrease of promoter activity compared with the C allele (*P* < 0.01) in human umbilical vein endothelial cells (HUVECs) [31]; as a result, variant genotypes CT and TT correlated with significantly lower levels of serum visfatin compared with genotype CC [21, 32]. The role of high expression of visfatin in various cancers have been identified by previous studies [33]. In general, overexpression of visfatin increased the activity of a number of signaling pathways that encourage carcinogenesis, such as NAD-dependent SIRTs, PI3K/Akt, ERK1/2, and STAT3 [33, 34]. The expression of visfatin has been shown to closely interact with inflammation and immune-related cytokines; for example, incubation with IL-6 in HUVECs induced a significant increase in visfatin mRNA levels [31]. With respect to -1535C>T, the serum levels of hs-CRP, IL-6, and TNF-α in CC carriers were significantly higher than those of TT carriers [21]. On the basis of previous research, the polymorphism of visfatin -1535C>T being related to HCC risk is biologically plausible.

The difference of the genotype frequency between the ethnic Zhuang and Han populations in this study was not statistically significant (*χ^2^*= 1.403, *P* = 0.496). However, after adjusting for confounding factors, we observed a significant association between visfatin -1535C>T polymorphism and HCC risk among the Zhuang population, but not among the Han population. We hypothesized the reason for this difference to be that HCC development may be jointly determined by both genes and the environment. Indeed, most of the ethnic Zhuang population in Guangxi live in rural areas or remote mountainous regions, where the major foods, such as rice, corn, and peanuts, are frequently contaminated by AFB1, which means that people in these regions have more environmental risk factor exposure. Similar conclusions have been reached before. Zeng *et al.* [35] found APE1-148 polymorphisms were associated with increased HCC risk among the Zhuang ethnicity, but not among the Han ethnicity. Long *et al.* [28] reported that HCC risk was significantly related to both risk genotypes and high-level or long-term AFB1 exposure in the Guangxi population. Therefore, we hypothesized that a gene may not necessarily predict HCC risk by itself, but instead it performed through a complex interaction of genes and the environment. In addition, our research showed a trend toward the CC genotype appearing among HCC patients of Han ethnicity; therefore, a relatively smaller sample size may be another reason for the inconsistent results.

We also observed a statistically significant association between -1535C>T polymorphisms and the risk of HBV-LC, and results indicated that the TT genotype reduced HBV-LC risk by nearly 40% compared with CC genotype (OR = 0.609, *P* = 0.047), However, the *P*-value was very close to 0.05. Moreover, no significant associations between -1535C>T polymorphisms and the risk of CHB were found in the present study, although the reduced trends of association were evident, as well as the comparison between CHB+LC and controls. Due to the relatively smaller sample sizes in the CHB and HBV-LC groups compared with the HCC group (*n* = 120; *n* = 140; and *n* = 243, respectively), the association between -1535C>T polymorphisms and the risk of CHB and HBV-LC are still uncertain. Therefore, the preliminary results found here must be confirmed by a larger study in the future. Additionally, the associations between visfatin -3187G>A polymorphisms and the risk of CHB, HBV-LC, and HBV-HCC were not statistically significant, therefore, the variants of visfatin -3187G>A did not contribute to HBV-related liver diseases in this population.

There were also no significant associations between the four constructed haplotypes, according to the observed genotypes of -1535C>T and -3187G>A, and the risk of CHB, HBV-LC, and HBV-HCC. Interestingly, Ooi *et al.* [26] found that -3187G>A and -1535C>T were at complete LD; however, our results showed that the LD between these two SNPs was very weak, which indicated that the contribution of genetic background may be distinct among different populations. Therefore, we further analyzed the genotype and allele frequencies among various populations, and our results confirmed this hypothesis. In fact, although the genotype frequency of -1535C>T loci were consistent with those in the Singapore population (*P*= 0.065) [26], the difference in the comparison of allele frequency was statistically significant (*P* = 0.027). Additionally, the genotype and allele frequencies of -1535C>T loci in the Guangxi population were significantly different from those in the Jinan, China population [24], and in the US population [25]. With respect to -3187G>A, our results were consistent with those of the HCB and the JPT populations, but were significantly different from those of the CEU and YRI populations. These results indicated that the polymorphisms of visfatin vary significantly among ethnicities. In fact, this ethnic variation factor was why we carried out this study in the Guangxi population: Guangxi is a multi-ethnic province in China.

Some limitations should be acknowledged in the present study. First, we studied only two SNPs in the visfatin promoter region, and other SNPs, such as rs2505568, rs9770242, rs1319501, and rs9034, require further study. Second, the study population was limited to the Zhuang and Han ethnicities. Therefore, these findings cannot be generalized to other ethnicities in Guangxi, such as the Yao, Miao, and Dong ethnicities. Third, as mentioned above, a relatively small sample size limited the statistical power in the analysis of this study, therefore, further studies with a larger sample size are required to confirm the results in different regions and races. Nevertheless, our study identified that visfatin-1535C>T polymorphisms were associated with reduced HBV-HCC risk, which suggests that this SNP loci may be used as a biomarker to estimate the risk of HCC in the Zhuang population. Our findings pave the way for HCC precision therapy, which is essential for controlling the HCC prevalence in Guangxi.

In summary, our results showed that visfatin -1535C>T polymorphisms were related to a significantly reduced risk of HBV-HCC, and the genotype TT and the allele T may be the protective factors for HBV-HCC among the ethnic Zhuang population in Guangxi, China.

## MATERIALS AND METHODS

### Subjects

All participants were recruited at the Third Affiliated Hospital of Guangxi University of Chinese Medicine in Liuzhou, China, during April 2015—January 2016. All participants gave written informed consent for this study, and this study was approved by the Ethics Committee of the Third Affiliated Hospital of Guangxi University of Chinese Medicine.

The study group consisted of 120 CHB patients, 140 HBV-LC patients, and 243 HBV-HCC patients. The inclusion of members of the CHB group were indicated by the following findings: elevated serum alanine aminotransferase (ALT) or aspartate aminotransferase (AST) levels (> 40 IU/mL); or HBV-DNA levels > 1,000 copies/mL. HBV-LC was diagnosed as described in our previous study [36]. The patient inclusions were primarily based on imaging examinations, through ultrasonography, magnetic resonance imaging, computed tomography, and pathological examination. HBV-HCC was diagnosed according to serum alpha fetal protein (AFP) levels (> 400 ng/mL) and an imaging examination, while some patients were further confirmed by pathological examination and underwent surgery. The exclusion criteria were patients who were co-infected with hepatitis A/C/D/E viruses; those who had other liver diseases, such as alcoholic liver diseases, or autoimmune hepatitis; or patients with familial aggregation.

Two hundred and twenty-four control subjects were randomly selected from the medical examination center of the same hospital. All patient controls were asymptomatic HBV carriers: that is, these patients were only hepatitis B surface antigen positive, while all the other laboratory tests, such as AST, ALT, AFP, HBV-DNA copies, and imaging examinations, were negative.

### DNA extraction and genotyping

Genomic DNA was extracted from 2 ml peripheral blood drawn from each subject by the standard phenol-chloroform method. The isolated DNA was stored at -80°C until analysis.

Visfatin gene polymorphisms were extracted by polymerase chain reaction (PCR), followed by a PCR-restriction fragment length polymorphism (PCR-RFLP) method. The following primers were used to amplify the PCR products: for -1535C>T, 5′TGTTTCAAACCTCGTTGCTGA-3′ and reverse 5′AGTGATGGTGGTGGTGGTA-3′; for -3187G>A, forward 5′AGCCCAGGATTTTGAGACCA-3′ and reverse 5′TCTGTGGATGAGGCCTTTCC-3′. The PCR cycling conditions were 95°C for 5 min, followed by 32 cycles at 95°C for 30 s, then applying annealing temperatures of 60°C for 45 s, 72°C for 1 min, and a final extension at 72°C for 10 min. Then, PCR products were digested overnight at 37°C with MvaI (for -1535C>T) and AcuI (for -3187G>A) restriction endonucleases, and further visualized in 3.0% agarose gel electrophoresis. On the basis of the respective allele size, the genotypes of visfatin-1535C>T were characterized as TT, CT, and CC, as well as for -3187G>A (Figure 1A, 1C).

To control the quality of genotyping, retesting occurred for any unclear results. Moreover, we randomly selected 10% of PCR products to validate the results from PCR-RFLP by employing a DNA sequencing analysis using an ABI Prism 3100 (Shanghai Sangon Biotech Co., Ltd., China), and a 100% concordance rate was achieved (Figure 1B, 1D).

### Statistical analysis

For each SNP, the Hardy-Weinberg equilibrium (HWE) was assessed by Pearson's goodness-of-fit Chi-square (*χ^2^*) statistic. The differences of qualitative characteristics, such as the distributions of gender, ethnicity, and genotypic frequencies, were analyzed using the *χ^2^* test. Continuous variables were expressed as the mean ±standard deviation (SD), and were tested by the one-way ANOVA test. Odds ratios (ORs) with 95% confidence intervals (CIs) were calculated for each allele in both the patient and control groups, using a binary logistic regressive analysis by adjusting for confounding factors, such as age, gender, smoking, drinking, and ethnicity.

Linkage disequilibrium (LD) analysis and haplotype analysis were performed using SHEsis software (http://analysis.bio-x.cn) [37]. The value of lowest frequency threshold for haplotype analysis was 0.03. Lewontin's *D'* and *r*^2^ were used to evaluate the LD between the two SNPs.

Statistical analysis was performed by SPSS 19.0 software (IBM Corporation, Armonk, NY, USA). All statistical tests were two-tailed, and a *P*-value < 0.05 was considered statistically significant.
